# Optimization of cultivation strategies for production of recombinant human papillomavirus type 58 major capsid protein L1 in *Hansenula polymorpha*

**DOI:** 10.1186/s40643-026-01059-8

**Published:** 2026-05-25

**Authors:** Natsima Kopitak, Wichittra Phimsen, Kittipol Sripui, Auntika Khunsom, Natchanon Pongsuwichedsak, Tatpong Boontawon, Thantawat Theeranan, Chuenchit Boonchird, Thunyarat Pongtharangkul

**Affiliations:** 1 Department of Biotechnology, Faculty of Science, Mahidol University, 272 Rama VI Road, Ratchathewi District, Bangkok, 10400 Thailand; 2 BioInnoTech, Faculty of Science, Mahidol University, Nakorn Pathom, Thailand

**Keywords:** HPV58 L1, *Hansenula polymorpha*, Virus-like particles, VLP, Bioreactor

## Abstract

Human papillomaviruses (HPVs) are a major cause of cervical cancer, which ranks fourth among cancers in women worldwide. A prophylactic vaccine composed of virus-like particles (VLPs) formed by the major capsid protein L1 of human papillomavirus (HPV) effectively prevents HPV infection but provides limited cross-protection against other HPV subtypes, highlighting the importance of multivalent prophylactic vaccines. In this study, cultivation conditions for recombinant HPV58 L1 protein production in the yeast *Hansenula polymorpha* were optimized to enhance growth and the volumetric yield of L1 protein. The results indicated that induction with methanol (at 1% v/v) was necessary for HPV58 L1 production in SYN6 medium, whereas a cultivation temperature of 37 °C was optimal for growth and production of HPV58 L1 protein. Subsequently, SYN6 medium supplemented with 10 g/L of either Hy-Express™ System II or HySoy was evaluated in batch and fed-batch bioreactor cultivations. Fed-batch cultivation with HySoy supplementation under a constant feeding rate achieved an OD_660_ of 117 (26.18 g-CDW/L), a volumetric L1 yield of 312 mg/L, and a productivity of 4.7 mg/L/h, which were significantly higher than those obtained with the control SYN6 medium (92 mg/L; 2.2 mg/L/h). These findings demonstrate that fed-batch cultivation with HySoy supplementation offers a practical and efficient strategy for HPV58 L1 production at the bioreactor scale.

**Graphical abstract:**

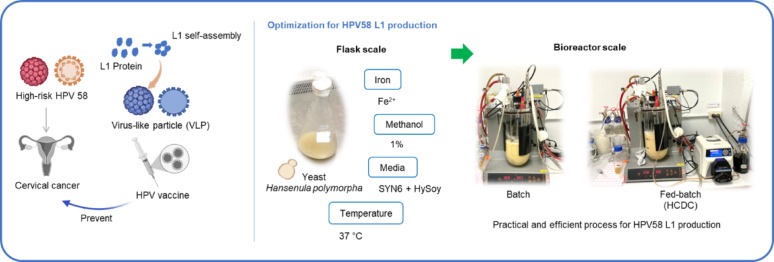

**Supplementary Information:**

The online version contains supplementary material available at 10.1186/s40643-026-01059-8.

## Introduction

HPV vaccine can prevent cervical cancer which is the fourth most common cancer in women worldwide. According to the 2023 World Health Organization’s (WHO) Report, about 600,000 incidences of cervical cancer in women with 340,000 female mortalities were expected annually (Bruni et al. 2023). Human papillomaviruses (HPVs), the member of Papillomaviridae family, are small non-enveloped icosahedral viruses (52–55 nm in diameter) with a circular, double-stranded DNA genome. The HPV capsid structure comprises of major capsid protein (L1) and minor capsid protein (L2). Pentameric L1 capsomeres can self-assemble into 72 L1 pentamers. An attachment of HPV virus to the host cell involves with the major capsid protein L1 whereas minor capsid protein L2 plays an essential role in an infectious process (Buck et al. 2013). Human papillomaviruses (HPVs) have more than 200 subtypes. In general, they can be categorized according to their oncogenic properties into (1) High-Risk (HR) types leading to cervical cancer (i.e., HPV16, 18, 31, 33, 35, 39, 45, 51, 52, 56, 58, 59, and 68), and (2) Low-Risk (LR) types detected in genital wart (i.e., HPV6 and 11) (Muñoz et al. 2003).

As the major capsid protein L1 contains highly immunogenic epitope, HPV vaccines in a form of virus-like particles (VLPs) of major capsid protein L1 have been developed and commercialized (Kirnbauer et al. 1992). The HPV vaccines derived from recombinant major capsid protein L1 VLPs include Cervarix™ (GlaxoSmithKline) and Gardasil^®^ (Merck & Co. Inc). Cervarix™ is a bivalent HPV vaccine (2vHPV) comprising of HPV 16 and 18 VLP produced by insect cells baculovirus. Gardasil^®^4, a quadrivalent HPV vaccine (4vHPV), comprised of L1 VLPs of HPV 6, 11, 16, and 18 produced by the yeast *Saccharomyces cerevisiae*. Although 70% of all cervical cancers was caused by HPV 16 and 18, they offered limited cross-protection against other HPV subtypes (Joura and Pils 2016). Therefore, multivalent HPV vaccines have been developed to target several high-risk (HR) HPV types. For example, Gardasil^®^9, a nanovalent HPV vaccine (9vHPV), comprises of L1 VLPs of HPV types 6, 11, 16, 18, 31, 33, 45, 52, and 58. The Food and Drug Administration (FDA) approved 9vHPV on December 10, 2014. It was cleared for use in females aged 9 to 26 and males aged 9 to 15 (Petrosky et al. 2015).

In 2017, the Thai Center for Disease Control and Prevention (CDC) included the HPV vaccine in the Expanded Program on Immunization (EPI). Therefore, every Thai female aged 11–12 years receives 2 doses of either the 2vHPV or 4vHPV vaccine (Bruni et al. 2023). Females with an age higher than 15 years old, on the other hand, would require 3 doses and currently are not included in the immunization program. Moreover, in East Asia, human papillomavirus types 52 and 58, not included in 2vHPV and 4vHPV vaccines, exhibit a uniquely high prevalence that distinguishes the region’s epidemiological profile from Western nations, where HPV16 and 18 typically dominate. Epidemiological studies in Northern and Southern China report that HPV52 is the most prevalent high-risk type (approx. 19%–23% of all infections), followed by HPV58 (approx. 13%–17%), often outperforming HPV16 in general screening programs (Shen et al. 2026). Similarly, in Thailand, reports from the WHO (WHO, 2025) and the ICO/IARC HPV Information Centre (ICO/IARC HPV Information Centre, 2023) indicate that while HPV16/18 cause approx. 67% of cancers in Thailand, HPV58 is the third or fourth most frequent oncogenic type across all lesion grades. In some regional Thai cohorts (e.g., Lampang), HPV58 and HPV52 combined are found in over 30% of high-risk infections. Meta-analyses confirm that the prevalence of HPV52 and 58 in East Asia is 3 to 5 times higher than the global average (Chan et al. 2014), necessitating the development of localized vaccine strategies (Xia et al. 2025; Yuan et al. 2024; Wang et al. 2024a, b), such as the Cecolin 9 as well as other regional 11-valent (NCT05262010) and 14-valent (SCT1000) candidates—specifically engineered to target the high prevalence of HPV52 and 58 in Asian cohorts.

The methylotrophic yeast *Hansenula polymorpha* has been used as an expression platform for the manufacture of recombinant proteins including vaccines, biopharmaceuticals, and enzymes for more than 30 years. *H. polymorpha* has unique characteristics and benefits including available strong promoters, accessible genetic tools, thermotolerance, an ability to utilize a variety of carbon sources (Manfrão-Netto et al. 2019). Recently, an 11-valent HPV vaccine candidate produced in *H. polymorpha* was developed by the National Vaccine and Serum Institute (NVSI) of China in association with Sinopharm. Data from Phase II/III clinical trials indicated that the vaccine is highly immunogenic, successfully inducing neutralizing antibodies against all 11 targeted HPV types, including HPV 6, 11, 16, 18, 31, 33, 45, 52, 58, 59, and 68 (Wang et al. 2024a, b; NVSI, 2025a, b). Although numerous studies have reported the expression and structural characterization of HPV L1 virus-like particles, comparatively few publications provide detailed bioprocess information such as volumetric yield, productivity, or fermentation performance under high-cell-density cultivation. In addition, production data for the NVSI 11-valent HPV vaccine remain proprietary and have not been publicly disclosed. Consequently, there is limited publicly available information on the production capacity of *H. polymorpha* for HPV L1 at the bioprocess level. The present study, therefore, aims to evaluate HPV L1 production in *H. polymorpha* under controlled fermentation conditions and to establish benchmark data for volumetric yield and production productivity.

While our previous report focused on production of HPV52 L1 (Phimsen et al. 2024), this study focused on optimizing the cultivation medium and conditions for HPV58 L1 production in *H. polymorpha*. The effect of iron supplementation and methanol induction were examined. To enhance both cell growth and L1 protein yield, several non-animal derived complex nitrogen sources were supplemented into SYN6 medium, a defined medium previously developed for cultivation of *H. polymorpha*. Moreover, we investigated how cultivation temperature influenced growth and L1 production as well as how cultivation temperature interacted with the cultivation medium. Furthermore, the cultivation conditions at a bioreactor scale including batch and fed-batch cultivation strategy with high cell density (HCDC) were optimized to maximize the L1 protein yield. Since the recombinant *H. polymorpha* strain used in our previous study on HPV52 L1 production and the strain used in the present study were constructed using the same host background and engineering strategy, comparing the results obtained from these two systems highlights the importance of strain-specific process optimization. These findings underscore the need for tailored bioprocess strategies for different recombinant strains and provide useful guidance for the large-scale production of HPV L1 and other recombinant proteins in *H. polymorpha*.

## Methods

### Microorganism and plasmid used

The recombinant *H. polymorpha* HPV58 was constructed based on *H. polymorpha* NCYC495 (SH4330) ura3 (Ura−) kindly provided by the Yeast Genetic Resource Center-National BioResource Project (YGRC-NBRP), University of Osaka, Japan. The plasmid pTB9 harboring L1 HPV58 (Fig. S1, Supplementary Materials) was constructed by substitution of OL-L1 HPV52 in the plasmid pTB6 (Phimsen et al. 2024) with OS-L1 HPV58 and introduced into the host strain (Boontawon et al. 2015). Selection and maintenance of the recombinant yeast were carried out following the same procedure as that described for *H. polymorpha* HPV 52 (Phimsen et al. 2024).

## Chemicals and media

Chemicals used were of analytical or molecular grade from recognized manufacturers including Difco (France), Kerry (USA), and LabM (UK). Antibodies and standard protein used were the same as those described for *H. polymorpha* HPV 52 (Phimsen et al. 2024).

## Inoculum preparation and flask-scale cultivation

Briefly, SC-Ura broth was used for preparation of pre-inoculum. Then, the inoculum grown in SYN6 medium supplemented with 1% (w/v) glycerol was used as an inoculum for flask-scale or bioreactor-scale cultivation. Detailed protocols on inoculum preparation and flask-scale cultivation are provided in Phimsen et al. (2024). All flask-scale cultivation experiments were performed in three independent biological replicates under identical conditions, except the experiments on effect of methanol concentration and nitrogen source supplementation. In addition, all analytical measurements were carried out in duplicate or triplicate (e.g., protein analysis) using standardized methods to ensure the reliability of the results.

## Effect of iron supplementation and methanol induction

Growth and HPV58 L1 protein production were compared in SYN6 medium composed of 2 forms of Iron supplementation. Ferrous (Fe^2+^) from 66.5 mg/L ammonium iron (II) sulfate hexahydrate and ferric (Fe^3+^) from 82.7 mg/L ammonium iron (III) citrate were evaluated separately in SYN6 medium. Inoculum (approximately 1–2% v/v) was transferred to obtain an initial OD_660_ of 0.1 and cultivated in 100 mL of SYN6 medium supplemented with 1.2% glycerol at 30 °C for 36 h (growth phase). For the induction phase, the medium was replaced with an equal volume of fresh SYN6, either with or without methanol supplementation (0, 0.25, 0.5, and 1%v/v methanol), and cultivation continued at 30 °C for 24 h. Suitable iron supplement and methanol concentration, giving high volumetric L1 yield, were selected for further experiments.

## Effect of non-animal derived nitrogen sources supplementation

To enhance growth and HPV58 L1 protein production, various non-animal derived nitrogen sources, including HyExpress™ System II, HyExpress™ System IV, HySoy, and HyYest412 (Kerry, USA) were supplemented into SYN6 medium at 10 g/L. The total nitrogen contents in SYN6 medium were 1.3, 0.98, 0.91, and 1.09 g/L, respectively. Cultivation was performed at 30 °C according to the method described for flask-scale cultivation as provided in Phimsen et al. (2024). The nitrogen source that supported good growth and high L1 protein production was subsequently selected for further evaluation.

### Effect of temperature on growth and L1 production

Effects of cultivation temperature (at 25, 30, and 37 °C) were investigated using SYN6 medium with and without supplementation of HySoy (at 10 g/L). The optimum temperature giving high L1 protein production was selected and applied further.

## Bioreactor-scale cultivation

Bioreactor-scale cultivation was performed in a 5 L bench-top bioreactor (Infors, Switzerland) at a working volume of 2 L for batch and 3 L (final volume) for fed-batch mode. Preparation of bioreactor and feed solutions were carried out following the same procedure as that described in Phimsen et al. (2024). In batch cultivation, SYN6 medium with and without supplementation of the selected non-animal derived nitrogen sources (i.e., HyExpress™ System II and HySoy) were evaluated. In fed-batch cultivation, both constant and exponential feeding were evaluated.

The cultivation was divided into 2 phases including growth phase (before methanol induction) and induction phase (during methanol induction). The cultivation parameters, including pH, %DO, temperature, and agitation speed, were monitored and recorded using IRIS software (version 5.3). The pH was maintained at 5.0 using 25% ammonia and 10% phosphoric acid solutions while the temperature was maintained at 37 °C. During batch cultivation, when glycerol was completely consumed as indicated by a rapid increase in %DO, methanol feeding (500 g/L) was initiated and regulated via DOT-stat control to maintain %DO at 10–30%. For fed-batch or high-cell density cultivation (HCDC), once the glycerol was completely consumed, the feed solution (400 g/L of glycerol and 200 g/L of HyExpress™ System II or HySoy) was added at a constant rate (10 mL/h) or at an exponential rate (Table S1, Supplementary Materials). The feeding was stopped when OD_660_ reached a value of 100. After that, when %DO rapidly increased again, methanol induction was initiated and maintained as previously described.

## Sample analysis

During cultivation, samples were collected every 6 h and analyzed for OD_660_, pH, cell dry weight (CDW), L1 protein (Western blot and Indirect ELISA) as well as residual glycerol and methanol (HPLC-RI) following the protocols described in Phimsen et al. (2024). The percentage of cells harboring HPV58 L1 gene was determined by PCR using primers designed specifically for amplification of a full expression cassette of HPV58 L1, with an expected product size of 2,471 bp (FMDp Eco-F: GAGAATTCAATGTATCTAAACGCAAACTC and MOXT Sma-R: CACCCGGGGATAT CACCACAACGTCC).

## Results

### Effect of iron supplementation and methanol induction

**Fig. 1:**
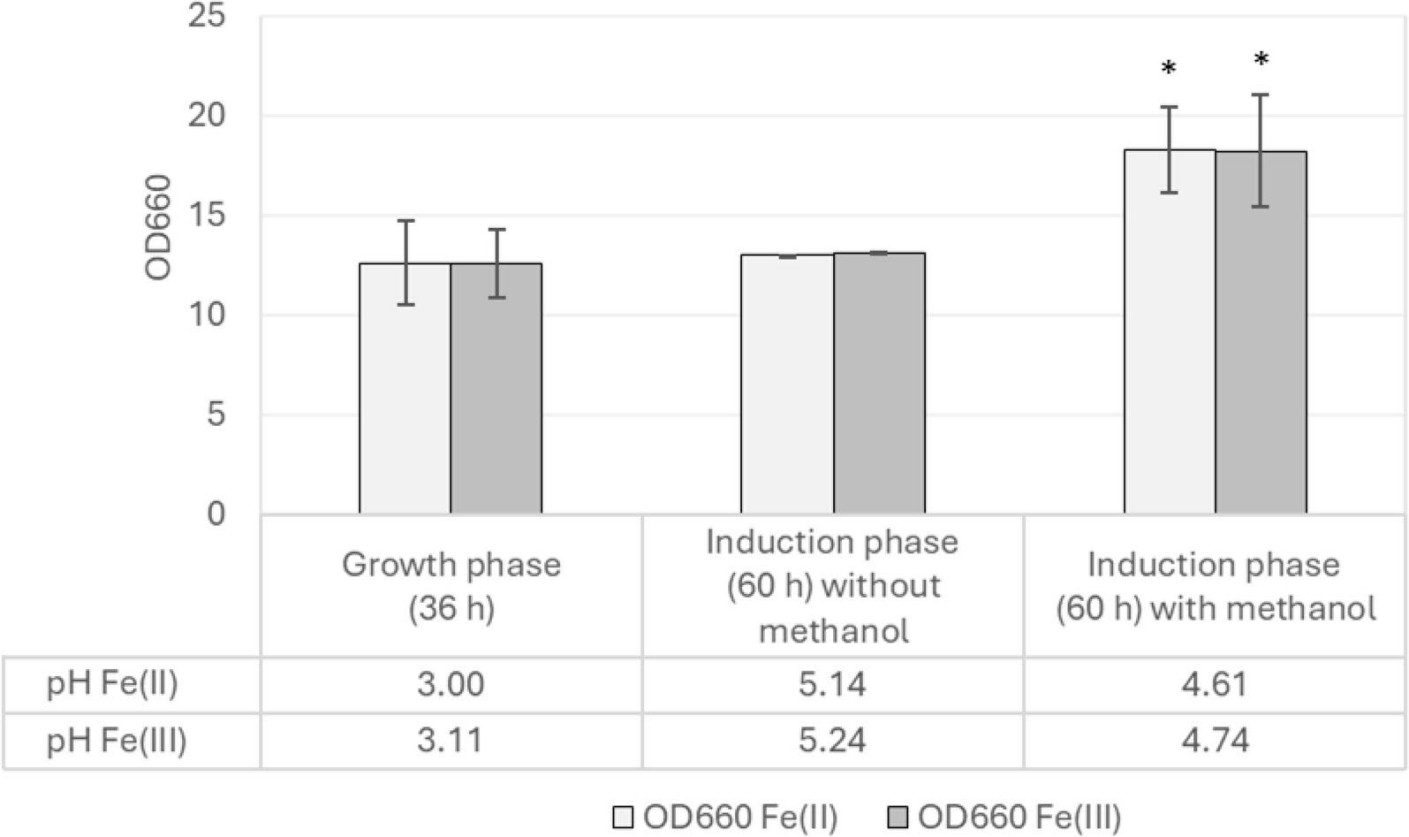
Effects of iron supplementation and methanol induction on pH and cell growth of the recombinant *H. polymorpha* HPV 58. Cells were cultivated in SYN6 medium supplemented with 1.2% glycerol and either ferrous (Fe^2+^; ammonium iron (II) sulfate hexahydrate) or ferric (Fe^3+^; ammonium iron (III) citrate) for 36 h (growth phase) before being transferred to SYN6 medium with or without methanol (induction phase). Bar graphs represent the mean values from three independent biological replicates, and error bars indicate standard deviations. Asterisks indicate statistically significant differences (*P* < 0.05) from the values shown in growth phase (control)

**Fig. 2:**
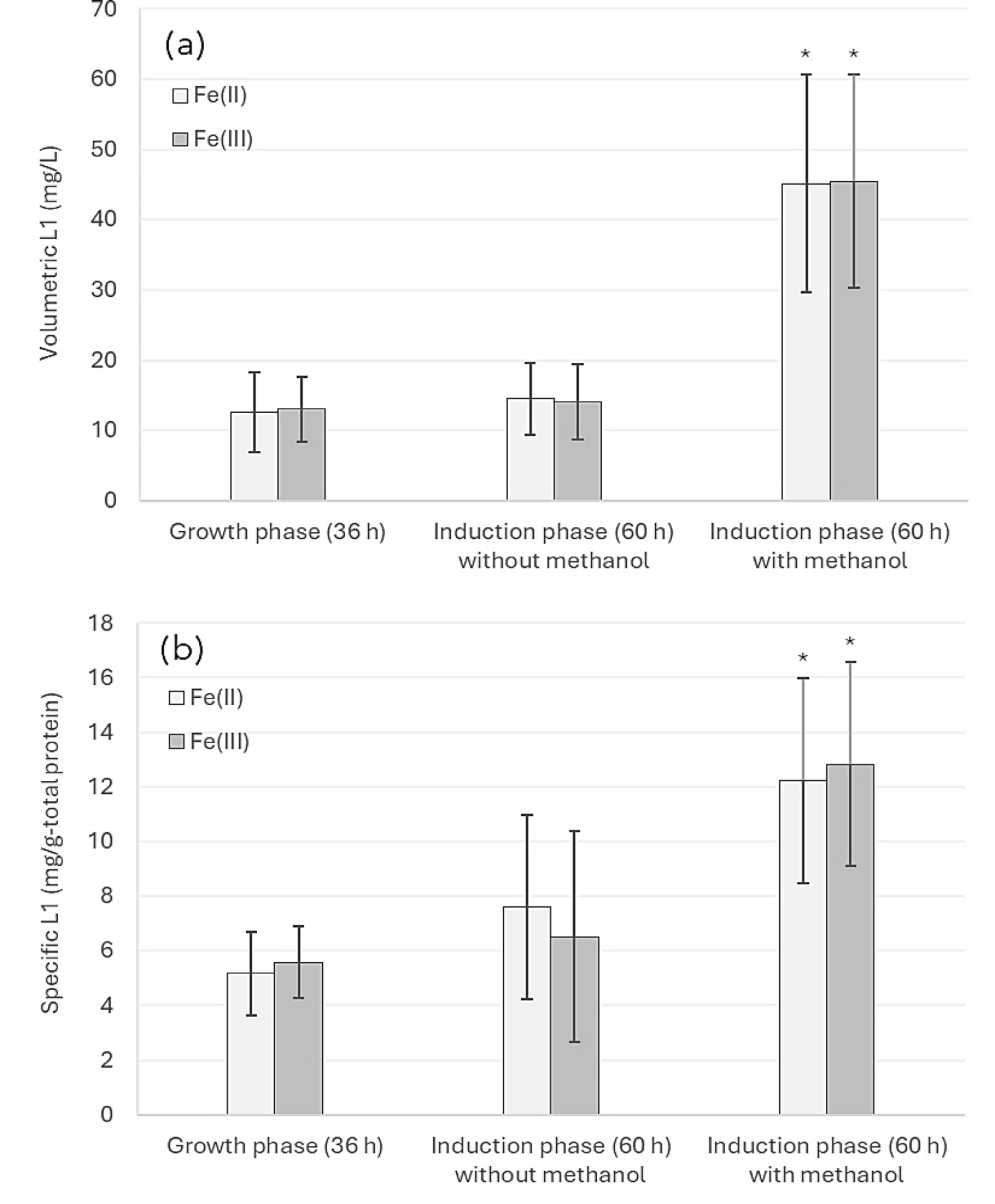
Effects of iron supplementation and methanol induction on L1 production of the recombinant *H. polymorpha* HPV 58;** a** L1 concentration in volumetric yield (mg/L) and** b** specific yield (mg/g-total protein). Cells were cultivated in SYN6 medium supplemented with 1.2% glycerol and either ferrous (Fe^2+^; ammonium iron (II) sulfate hexahydrate) or ferric (Fe^3+^; ammonium iron (III) citrate) for 36 h (growth phase) before being transferred to SYN6 medium with or without methanol (induction phase). Bar graphs represent the mean values from three independent biological replicates, and error bars indicate standard deviations. Asterisks indicate statistically significant differences (*P* < 0.05) from the values shown in growth phase (control)

Iron is one of the micronutrients in the defined minimal mineral medium SYN6 used for the cultivation of *H. polymorpha*. The original SYN6 medium (Jenzelewski 2002) provided ferrous iron (Fe^2+^) in the form of ammonium iron (II) sulfate hexahydrate. However, other researchers modified the SYN6 medium and instead provided ferric iron (Fe^3+^) in a form of ammonium iron (III) citrate (personal communication). In this study, ferric iron (Fe^3+^) from ammonium iron (III) citrate and ferrous iron (Fe^2+^) from ammonium iron (II) sulfate hexahydrate were evaluated to investigate the effects of iron supplementation and methanol induction on HPV58 L1 protein production. During the growth phase, cells were cultivated in SYN6 medium supplemented with 1.2% glycerol for 36 h. Subsequently, during the induction phase, the medium was replaced with SYN6 with or without methanol supplementation to evaluate the effect of methanol. OD_660_ and pH values are presented as averages of three independent experiments. No statistically significant differences were observed between the two iron supplements in terms of cell growth, pH, and L1 protein production (Figs. 1 and 2). Therefore, ferrous iron (Fe^2+^) in the form of ammonium iron (II) sulfate hexahydrate was selected as the iron source for SYN6 medium due to its lower cost and commercial availability in bulk.

**Fig. 3:**
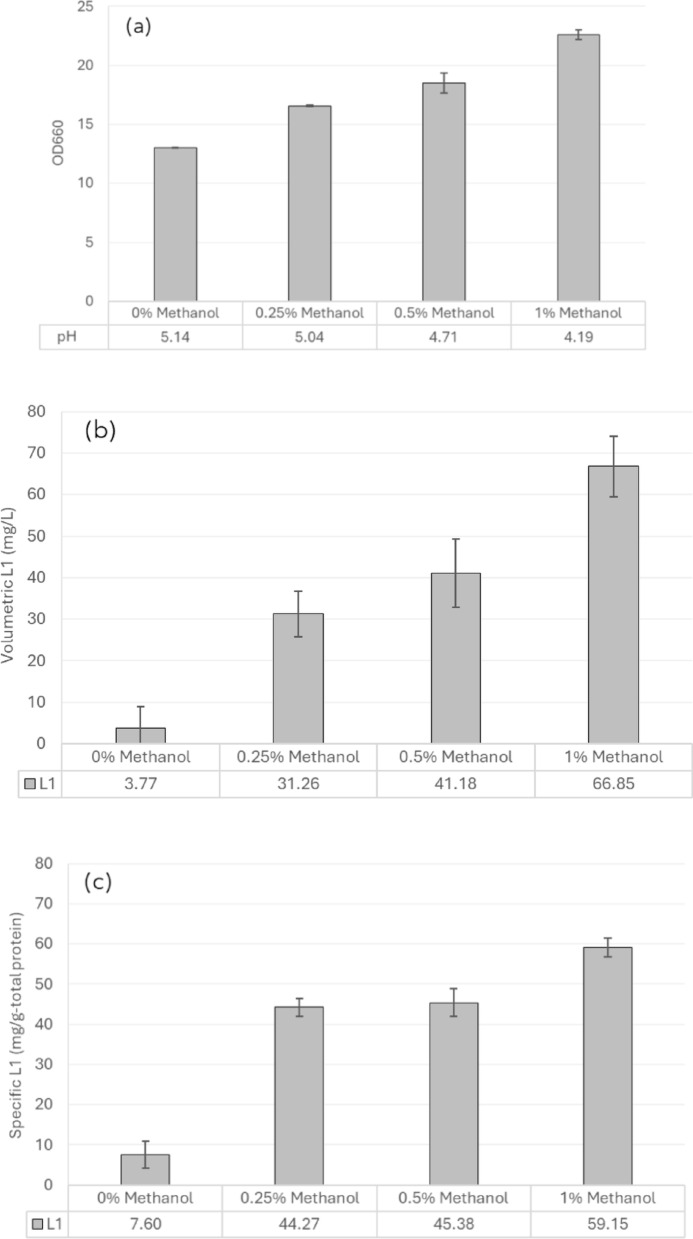
Effects of methanol concentration on cell growth and L1 production of the recombinant *H. polymorpha* HPV 58;** a** OD_660_ and pH,** b** volumetric L1 yield (mg/L), and** c** specific L1 yield (mg/g-total protein). Cells were cultivated in SYN6 medium supplemented with 1.2% glycerol and ammonium iron (II) sulfate hexahydrate for 36 h (growth phase) before being transferred to SYN6 medium with various concentrations of methanol (0, 0.25, 0.5, and 1% v/v). Bar graphs represent the mean values from two independent biological replicates, and error bars indicate standard deviations

In *H. polymorpha*, methanol serves as both carbon source and inducer of recombinant protein production. However, recombinant protein expression under methanol-free or derepressed conditions has also been reported. To investigate whether methanol induction is required for HPV L1 production in the recombinant strain used in this study, in this study, methanol concentrations in the range of 0–1% (v/v) were evaluated for their effects on growth and HPV 58 L1 protein production. The results indicated that SYN6 medium supplemented with 1% methanol gave the highest biomass and L1 production, both in terms of volumetric yield (mg/L) and specific yield (mg/g-total protein) (Fig. 3). The optical density (OD_660_) increased gradually with higher methanol concentrations, from 12.98 at 0% methanol to 22.60 at 1% methanol. The culture pH slightly decreased with increasing methanol concentration, dropping from 5.14 at 0% to 4.19 at 1% methanol (Fig. 3a). Volumetric L1 yield increased markedly from 3.77 mg/L in cultures without methanol induction to 66.85 mg/L at 1% methanol (Fig. 3b), while the specific L1 yield increased from 7.60 mg/g-total protein to 59.15 mg/g-total protein under the same conditions (Fig. 3c). As the cultivation with 1% methanol showed higher biomass and L1 production than other conditions, therefore, an induction with 1% methanol was employed further.

### Effects of non-animal-derived nitrogen source supplementation

**Fig. 4:**
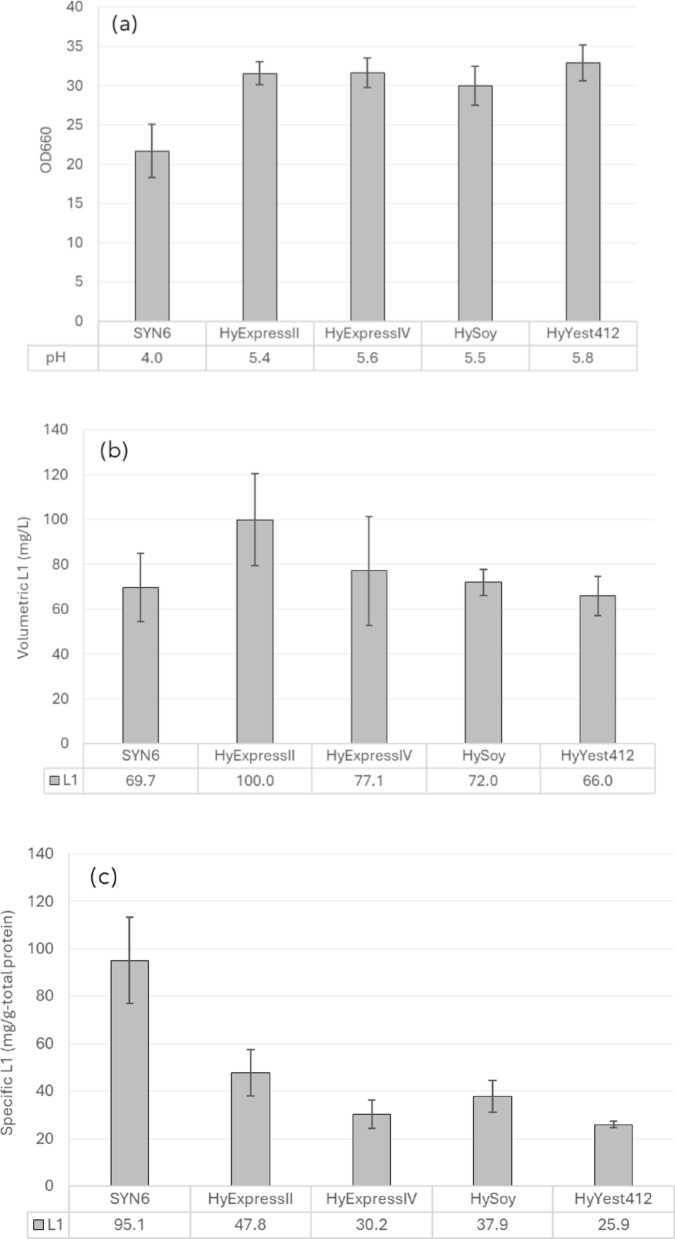
Effect of non-animal derived nitrogen sources supplementation;** a** OD_660_ and pH,** b** volumetric L1 yield (mg/L), and** c** specific L1 yield (mg/g-total protein). Recombinant *H. polymorpha* HPV 58 was cultivated in SYN6 medium supplemented with either Hy-Express™ System II, Hy-Express™ System IV, HySoy, or Hy-Yest 412 for 36 h (growth phase) before being transferred to the same medium with 1% methanol (induction phase). Bar graphs represent the mean values from two independent biological replicates, and error bars indicate standard deviations

SYN6 medium supplemented with ferrous iron was further supplemented with four different non-animal-derived nitrogen sources; Hy-Express™ System II, Hy-Express™ System IV, HySoy, and Hy-Yest 412, which were expected to promote growth and recombinant L1 protein production. Growth and pH after a methanol induction are shown in Fig. 4a. Improvement in growth and recombinant L1 production were observed in all media after 24 h of methanol induction. Supplementation with complex nitrogen sources helped maintain the culture pH at around 5.4–5.8, which is the optimal pH for the growth of *H. polymorpha*. SYN6 medium without complex nitrogen source supplementation showed a decrease in pH, possibly because of continuous acidification associated with an ammonia uptake via an ammonia/H^+^ antiport in *H. polymorpha* (Jenzelewski 2002). Unlike the pattern reported for recombinant *H. polymorpha* HPV52 (Phimsen et al. 2024), supplementation with a complex nitrogen source did not improve the volumetric L1 yield (Fig. 4b) and instead led to a notable decrease in the specific L1 yield (Fig. 4c). Nonetheless, since growth and product formation can vary substantially during scale-up from flask to bioreactor, two different nitrogen sources (Hy-Express™ System II and HySoy) were selected for further evaluation in bioreactor-scale cultivation under batch and fed-batch strategies. Hy-Express™ System II was selected based on its high volumetric and specific L1 yields, while HySoy was chosen for its cost-effectiveness and its low variation in L1 yield.

### Effect of cultivation temperature on cell growth and HPV58 L1 protein

**Fig. 5:**
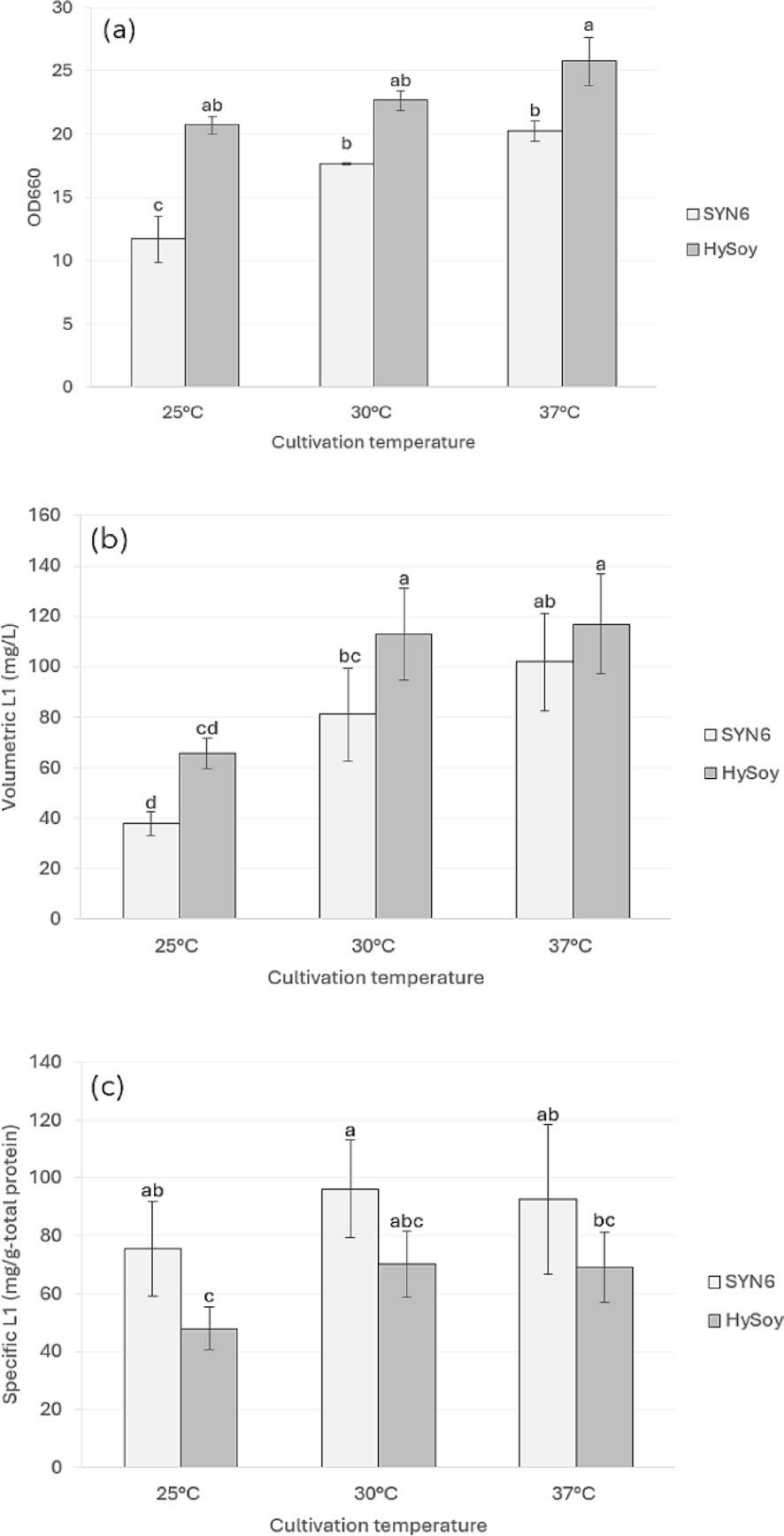
Effect of cultivation temperatures (25, 30, and 37 °C) on cell growth and L1 production of the recombinant *H. polymorpha* HPV 58;** a** OD_660_,** b** volumetric L1 yield (mg/L), and** c** specific L1 yield (mg/g-total protein). Cells were cultivated in SYN6 medium supplemented with 10 g/L HySoy for 36 h (growth phase) before being transferred to the same medium with 1% methanol (induction phase). Bar graphs represent the mean values from three independent biological replicates, and error bars indicate standard deviations. Different alphabets above the bars indicate statistically significant differences among treatments (*P* < 0.05) based on Tukey’s multiple comparison test

To assess the effect of temperature on growth and HPV58 L1 production, SYN6 supplemented with HySoy was used, and the results were compared with those obtained using the original SYN6 medium as a control. Temperature significantly affected cell growth (*P* = 0.002), volumetric L1 yield (*P* < 0.001), and specific L1 yield (*P* = 0.002), with higher temperatures leading to increased values in all parameters. HySoy supplementation also had a significant effect on growth (*P* < 0.001), volumetric L1 yield (*P* = 0.001), and specific L1 yield (*P* < 0.001). Across all temperatures tested, HySoy supplementation improved growth and volumetric L1 yield, but resulted in a reduction in specific L1 yield (Fig. 5). The results indicated that a cultivation temperature at 37 °C was most suitable for HPV58 L1 production, offering the highest L1 yield and improved cost-effectiveness due to reduced heat removal requirements in large-scale processes.

### Bioreactor-scale cultivation under batch and fed-batch (high cell density) cultivation

Two different modes of cultivation, batch and fed-batch (high cell density or HCD) cultivation were compared in this study. Batch cultivation with low cell density was carried out without nutrient feeding during the growth phase, while HCD cultivation (HCDC) was fed with glycerol and selected nitrogen sources, HyExpress™ System II or HySoy, using either a constant or exponential feeding strategy. Methanol induction was subsequently controlled by cascade control based on dissolved oxygen (%DO) value (DOT-stat).

**Fig. 6:**
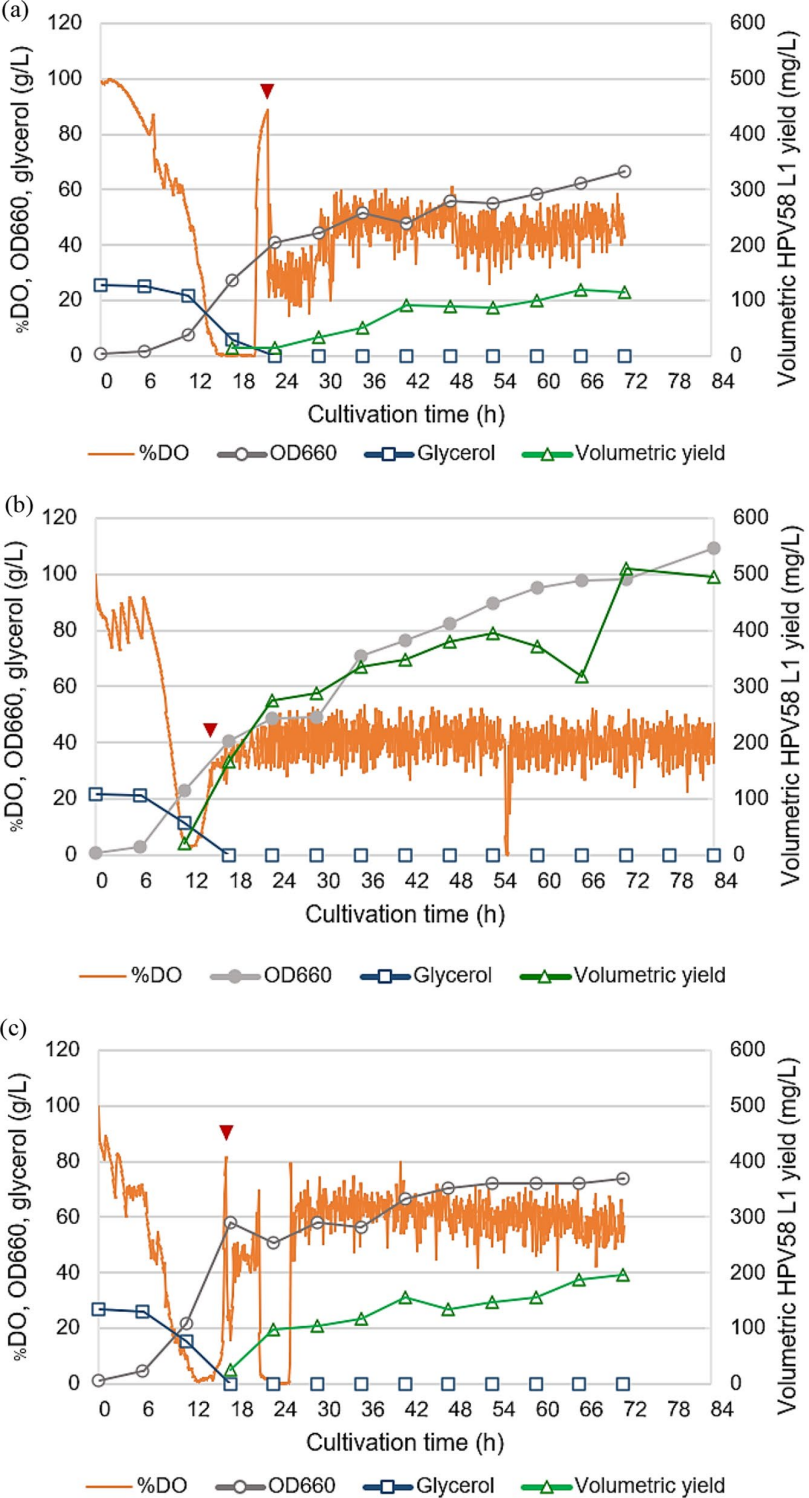
Batch cultivation using three different SYN6-based media:** a** SYN6,** b** SYN6 supplemented with Hy-Express™ System II, and** c** SYN6 supplemented with HySoy. Growth (OD_660_), % dissolved oxygen (%DO), glycerol concentration, and volumetric HPV58 L1 yield are shown. Inverted triangles indicate the start of methanol induction

In batch fermentation, *H. polymorpha* HPV58 was cultivated in SYN6 supplemented with 20 g/L glycerol and 10 g/L of the selected nitrogen source (Hy-Express™ System II or HySoy). During the growth phase at high carbon availability, high oxygen consumption was observed as indicated by a steady decrease in dissolved oxygen (%DO) (Fig. 6a–c). After 16–18 h, dissolved oxygen levels dropped to nearly zero, indicating glycerol depletion. Subsequently, %DO increased rapidly, and methanol induction via DOT-stat cascade control was initiated.

**Fig. 7:**
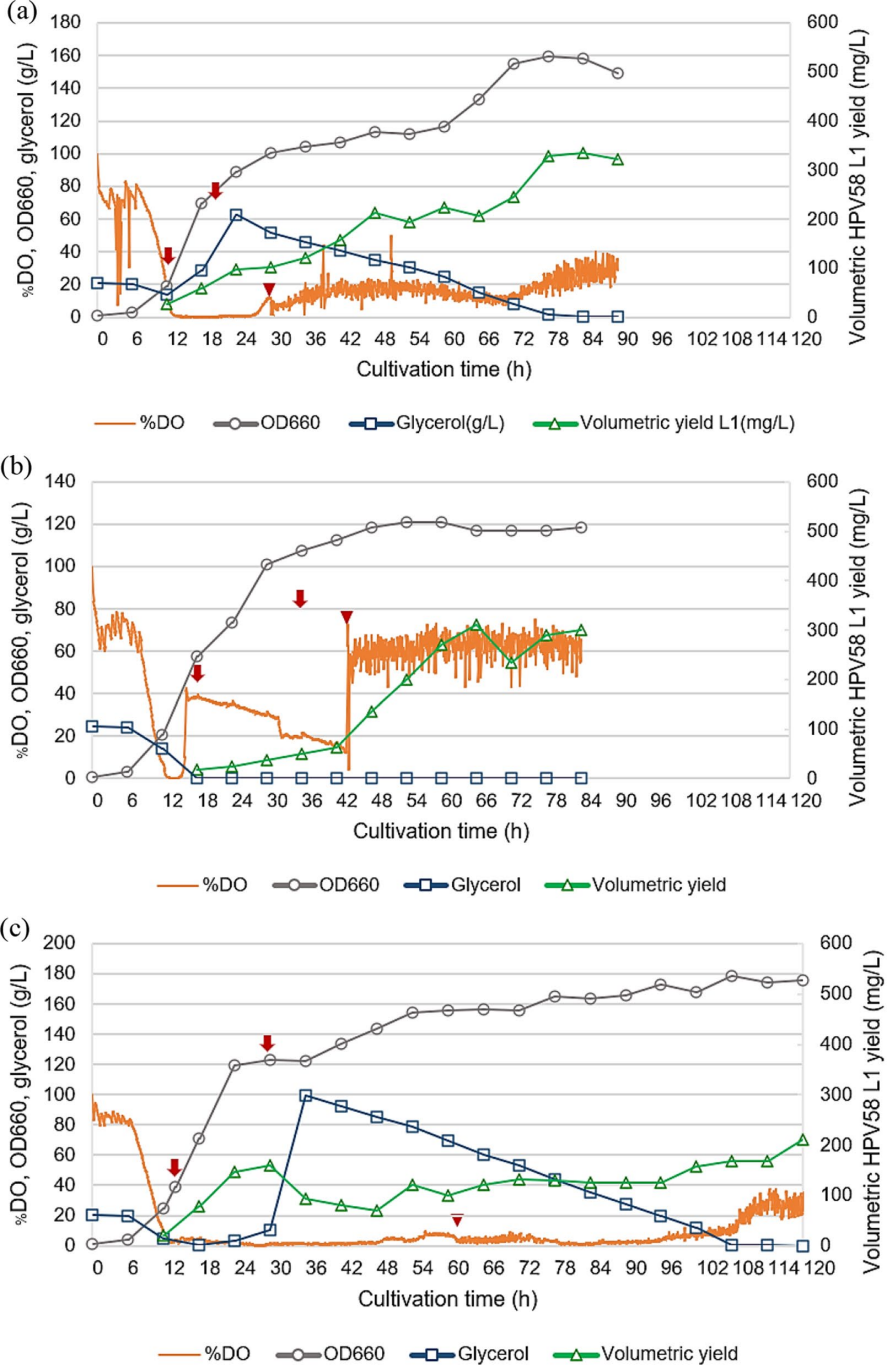
Fed-batch cultivation using three different SYN6-based media:** a** SYN6 supplemented with Hy-Express™ System II under exponential feeding,** b** SYN6 supplemented with HySoy under constant feeding, and** c** SYN6 supplemented with HySoy under exponential feeding. Growth (OD_660_), % dissolved oxygen (%DO), glycerol concentration, and volumetric HPV58 L1 yield are shown. Inverted arrows indicate the start and stop of nutrient feeding, whereas inverted triangles represent the start of methanol induction

In batch cultivation, complete glycerol consumption, indicated by a rapid increase in %DO, was observed at different cultivation time; 24 h for SYN6, and 18 h for SYN6 supplemented with Hy-Express™ System II or HySoy. The shorter induction time in the nitrogen source-supplemented media suggests that nitrogen-source supplementation promoted faster cell growth, leading to earlier glycerol depletion (Fig. 6a–c). At their respective optimal harvest periods, determined based on the maximum volumetric L1 yield, OD_660_ values of 48 for SYN6 (42 h), 98 for SYN6 with Hy-Express™ System II (72 h), and 67 for SYN6 with HySoy (42 h) were obtained. Similar to the trend observed in flask-scale (Fig. 4a), supplementation of complex nitrogen sources enhanced cell growth significantly. A consistent trend was observed for volumetric L1 yield: Hy-Express™ System II supplementation gave the highest volumetric L1 yield (510 mg/L), followed by HySoy (155 mg/L), and SYN6 (92 mg/L). Given that the increase in volumetric yield was associated with higher biomass levels, it is plausible that further enhancement of volumetric L1 yield could be achieved through HCDC. In terms of productivity, Hy-Express™ System II supplementation exhibited the highest performance (7.1 mg/L/h), whereas HySoy and SYN6 supplementation yielded 3.7 and 2.2 mg/L/h, respectively (Fig. 7). Therefore, a fed-batch cultivation strategy was subsequently applied to further improve HPV58 L1 yield and productivity.

In fed-batch cultivation, SYN6 medium was individually supplemented with 10 g/L of either Hy-Express™ System II or HySoy as nitrogen sources. When glycerol was completely consumed (indicated by an increasing trend of %DO), feed solution (400 g/L of glycerol with 200 g/L of Hy-Express™ System II or HySoy) was fed at an exponential rate for Hy-Express™ System II. The decision to prioritize exponential feeding for the Hy-Express™ System II was based on its superior performance during initial batch trials. Given that SYN6 + HyExpress II (Batch) yielded the highest volumetric L1 (510 mg/L) and productivity (7.1 mg/L/h) among all batch conditions, we focused on the feeding strategy most likely to maximize biomass and protein expression to justify the higher cultivation cost. In contrast, both constant and exponential feeding strategies were evaluated for HySoy. A constant feeding strategy was specifically selected to investigate the effect of a sub-maximal growth rate on HPV L1 production, as exponential feeding of HySoy resulted in the highest cell growth but the lowest overall productivity (1.6 mg/L/h).

**Fig. 8:**
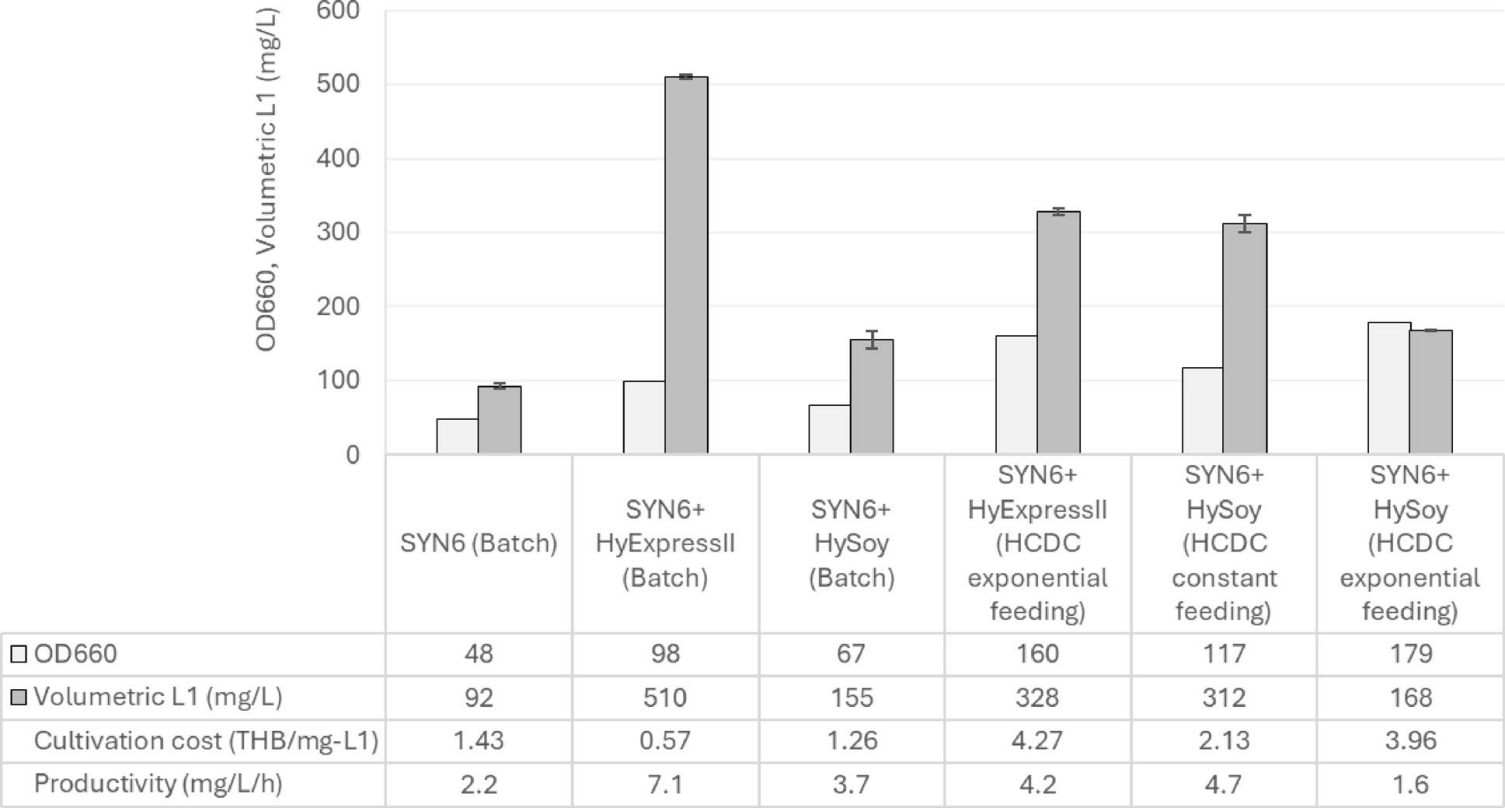
Growth, HPV58 L1 production, cultivation cost, and productivity from various cultivation conditions evaluated in this study. Cultivation costs are expressed as THB per mg of L1 protein and were calculated based solely on raw media components, excluding labor and overhead costs

The start and stop times of nutrient feeding and methanol induction are indicated (Fig. 8). The optimal harvest periods for the 3 conditions evaluated, Hy-Express™ System II (exponential feeding), HySoy (constant feeding), and HySoy (exponential feeding), were 78, 66, and 108 h, respectively. Among the fed-batch cultivations, Hy-Express™ System II (exponential feeding) gave the highest amount of HPV58 L1 protein at 328 mg/L, with an OD_660_ of 160 and a productivity of 4.2 mg/L/h. However, this was also associated with the highest production cost, at 4.27 THB/mg-L1. HySoy (constant feeding) gave a slightly lower yield at 312 mg/L and OD_660_ of 117 but resulted in the highest productivity at 4.7 mg/L/h and the lowest production cost at 2.13 THB/mg-L1, indicating that it was the most cost-efficient alternative among the fed-batch (HCDC) cultivations. Interestingly, although HySoy under exponential feeding achieved the highest cell density (OD660 = 179), the L1 protein yield was the lowest (168 mg/L), with a productivity of only 1.6 mg/L/h and a relatively high cultivation cost (3.96 THB/mg-L1). These results indicate that higher biomass levels did not always translate into higher HPV 58 L1 protein production.

**Fig. 9:**
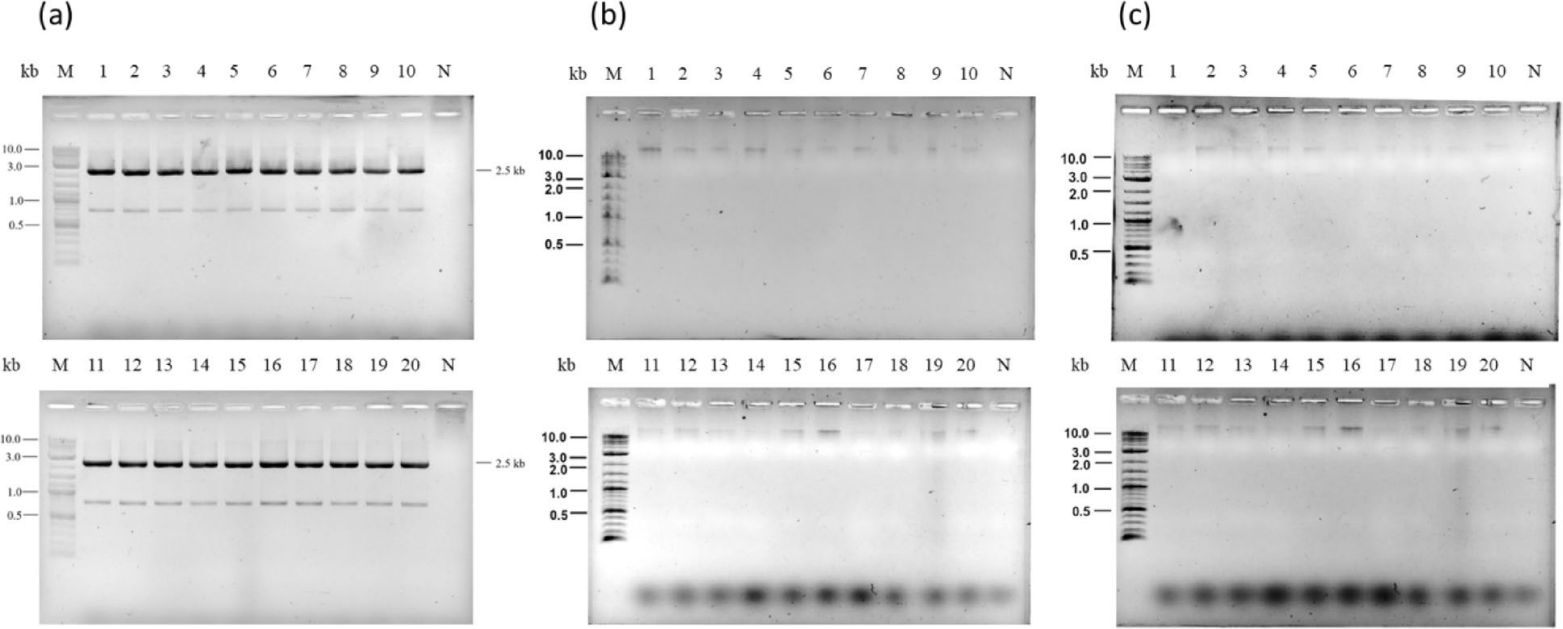
Colony PCR of the recombinant *H. polymorpha *recovered after (**a**) 120 h of fed-batch cultivation in SYN6 supplemented with HySoy,** b** 84 h of batch cultivation in SYN6 supplemented with Hy-Express™ System II, and** c** 120 h of fed-batch cultivation in SYN6 supplemented with Hy-Express™ System II. Lane M: 2-Log DNA Ladder (NEB, USA); Lane No.1-20: PCR product from the recombinant yeast; Lane N: Negative control

PCR analysis performed after batch and fed-batch cultivation revealed loss of an expression cassette in *H. polymorpha* cultivated using SYN6 supplemented with Hy-Express™ System II (Fig. 9b and c). In contrast, no gene loss was detected in SYN6 supplemented with HySoy (Fig. 9a), indicating stable maintenance of the expression cassette. Along with its high productivity and cost-effectiveness, HySoy appears to be a more suitable complex nitrogen source for HPV58 L1 protein production. Accordingly, fed-batch cultivation using SYN6 supplemented with HySoy under a constant feeding rate was selected as the optimal strategy for HPV58 L1 protein production.

## Discussions

### Role of iron supplementation and methanol induction

*Hansenula polymorpha* is a methylotrophic yeast widely used for recombinant protein production due to its available genetic tools, strong promoters, and ability to utilize various carbon sources in defined media (Manfrão-Netto et al. 2019). SYN6, a defined minimal mineral medium developed for the cultivation of *H. polymorpha*, includes a salt mixture, calcium chloride, microelements, vitamins, and trace elements that support high-cell-density cultivation (up to 100 g/L biomass) (Degelmann 2002). As iron is an essential micronutrient for yeast metabolism, several studies have investigated the effects of different iron salts on yeast physiology. For instance, studies on iron uptake by *Saccharomyces cerevisiae* have shown that various iron salts, including ferric EDTA, ferric citrate, ferrous chloride, and ferrous sulphate, exhibit no significant differences in biomass formation. Among these, ferrous sulphate is recommended because of its low cost, efficacy, and stability (Gaensly et al. 2014).

To evaluate the effect of iron on cell growth and HPV58 L1 protein production, the original SYN6 formulation, which provided ferrous iron (Fe^2+^) in the form of ammonium iron (II) sulfate hexahydrate (Degelmann 2002), was compared with a modified SYN6 medium containing ferric iron (Fe^3+^) in the form of ammonium iron (III) citrate. The chemical form of iron is considered an important factor affecting its bioavailability. Under aerobic conditions, iron mainly exists as ferric (Fe^3+^), a poorly soluble and biologically inaccessible form. Yeasts generally acquire iron through two major systems: the reductive pathway, in which ferric ion (Fe^3+^) is reduced to ferrous ion (Fe^2+^) before being transported via high-affinity Fe^2+^ transporters, and the siderophore-mediated pathway, where Fe^3+^ is chelated by organic ligands such as siderophores to form soluble complexes that facilitate cellular uptake (Lesuisse et al. 1987; Ramos-Alonso et al. 2020).

In *S. cerevisiae*, high-affinity Fe^2+^ uptake is mediated by the Fet3p-Ftr1p complex, which functions downstream of Fe^3+^ reduction by plasma membrane-bound ferric reductase (Askwith et al. 1994; Hassett et al. 1998). Although the corresponding transporters in *H. polymorpha* have not been fully characterized, the presence of genes encoding Fet3/Ftr1-like proteins suggests a conserved reductive iron uptake mechanism. A similar system has also been described in *Pichia pastoris*, another methylotrophic yeast, where a Fet3 homolog has been cloned and characterized, further supporting the conservation of high-affinity iron uptake systems among yeast species (Paronetto et al. 2001). *H. polymorpha* was originally isolated from soil (Manfrão-Netto et al. 2019), an environment where siderophore-mediated iron competition is high (Gu et al. 2020). It is plausible that *H. polymorpha* retains cryptic genes for opportunistic siderophore uptake, reflecting its evolutionary adaptation, a strategy well-documented in other yeasts such as *S. cerevisiae* (Stanford and Voigt 2020). However, current genomic and biochemical data show no evidence of endogenous siderophore biosynthesis in *H. polymorpha* or its synonym *Pichia angusta* (Wang et al. 2009).

In this study, no significant differences in OD_660_, pH, or volumetric HPV58 L1 yield were observed between Fe^2+^ and Fe^3+^ supplementation in SYN6 medium (Fig. 1), suggesting that *H. polymorpha* can efficiently reduce Fe^3+^ to Fe^2+^ prior to an uptake. Considering the comparable efficacy, cost, and availability, ammonium iron (II) sulfate hexahydrate was selected as the preferred iron source for SYN6 medium.

During the growth phase, *H. polymorpha* was cultivated in SYN6 medium containing 1.2% glycerol as a non-inducing carbon source to promote cell growth before induction. The HPV58 L1 gene was expressed under the control of the formate dehydrogenase (*FMD*) promoter, a strong methanol-inducible promoter belonging to the methanol utilization (MUT) pathway of *H. polymorpha*. Promoters in the MUT pathway are typically tightly regulated - repressed in the presence of glucose/glycerol and strongly induced in the presence of methanol (Suppi et al. 2013).

A study on transcriptomic profile of *H. polymorpha* CBS4732 (also known as CCY38-22-2, ATCC34438, or NRRL-Y-5445) under glucose- and methanol-supplemented growth conditions by van Zutphen et al. (2010) revealed that methanol dissimilation genes, including *MOX* and *FMD*, were activated by de-repression upon carbon source limitation or depletion rather than the presence of methanol, unlike genes of peroxisome biogenesis or proliferation which were induced by methanol. Therefore, many *H. polymorpha*-based industrial fermentation processes (e.g., hirudin, saratin, aprotinin, and phytase) were developed based on a de-repression of carbon sources (glucose or glycerol) without a methanol induction (Mayer et al., 1999; Manfrão-Netto et al. 2019). This powerful de-repression mechanism is a unique feature of *H. polymorpha* compared to other methylotrophic yeasts like *P. pastoris*. In *H. polymorpha*, promoters such as *MOX* and *FMD* can be activated to approximately 60–70% of their maximum methanol-induced levels upon depletion of a repressing carbon source, such as glycerol or glucose. In contrast, the primary *AOX*1 promoter in *P. pastoris* exhibits only minimal de-repression, reaching about 2–4% under similar conditions (Vogl and Glieder 2013). Therefore, previous studies have suggested that methanol induction is essential for achieving high-level recombinant protein production in *P. pastoris*, but not in *H. polymorpha*.

To our knowledge, the only industrial fermentation process that has successfully employed methanol induction in *H. polymorpha* is the production of hepatitis B surface antigen (HBsAg) produced as a particle with the recombinant antigen inserted into the host-derived membrane (Stöckmann et al. 2009). This process employs engineered *H. polymorpha* strains mainly derived from two parental lines, CBS4732 and DL-1, with the industrial strain RB11 (a CBS4732 derivative) and its subsequent derivatives have become a key platform for commercial production of several hepatitis B vaccines. Higher HBsAg upon methanol induction was, however, expected as a result of increased membrane proliferation.

In our study, an induction with methanol always resulted in significantly higher expression of L1 protein in *H. polymorpha* compared to a similar cultivation without methanol induction. The gradual increase in OD_660_ and decrease in pH along with higher methanol concentrations (Fig. 3a and b) is consistent with the increased metabolic activity and accumulation of organic acids during methanol induction. Interestingly, while the volumetric yield of L1 protein increased proportionally with methanol concentration, the specific yield plateaued at 0.25–1% methanol, indicating that methanol levels above 0.25% mainly promoted biomass accumulation rather than further improving the expression level. This observation highlights the importance of optimizing methanol concentration for both growth and L1 production in *H. polymorpha*. Despite the de-repression-based expression trend previously reported for CBS4732-derived strains, methanol induction was still required for the production of HPV58 L1 protein in *H. polymorpha* (based on *H. polymorpha* NCYC495). Our finding is consistent with a study reported by Suppi et al. (2013) in which 1% methanol was the most optimal for recombinant protein production in *H. polymorpha.* This observation agrees with many studies demonstrated that methanol induction was critical for achieving high protein expression (Liu et al. 2015; Xu et al. 2014), while non-induced cultures, which relied on de-repression, exhibited lower yields.

### Influence of non-animal-derived nitrogen sources

Since *H. polymorpha* can grow in both complex and defined media, evaluating the influence of nitrogen sources on cell growth and recombinant protein yield is valuable for optimizing cultivation conditions. For vaccine production, using non-animal-derived components free of potential antigens and prions is essential for product safety and regulatory acceptance. In this study, a cultivation medium based on the defined SYN6 formulation was supplemented with four different non-animal-derived nitrogen sources (Hy-Express™ System II, HyExpress™ System IV, HySoy, and Hy-Yest 412) selected for their pharmaceutical grade quality and compliance with cGMP standards. Detailed compositions of nitrogen sources are provided in Table S2 (Supplementary Materials).

The effect of complex nitrogen sources on recombinant protein production in *H. polymorpha* has been previously investigated. For instance, several types of peptones have been shown to enhance the production of recombinant parathyroid hormone (rPTH) fragment 1–34, with only minor effects on cell growth (Mueller et al. 2013). In addition, HyExpress™ System IV and HySoy were reported to improve the volumetric yield (mg/L) and specific yield (mg/g total protein), respectively, of HPV52 L1 protein, with no significant effect on growth in the recombinant *H. polymorpha* strain constructed using the same host and methodology as in this study. In contrast to those studies, our results in flask-scale cultivation showed comparable volumetric yield (mg/L) of HPV 58 L1 protein compared to the original SYN6 medium, although a slight increase in OD_660_ was observed (Fig. 4). The observed differences may result from variations in the target recombinant protein and the composition of the nitrogen sources, both of which can influence cellular nitrogen utilization and methanol metabolism during protein production. However, such effects may not be fully observable in small-scale cultivation, where pH and aeration cannot be precisely controlled. Therefore, bioreactor-scale cultivation should be performed to more accurately assess the difference.

Overall, the effects of increasing methanol concentration and supplementation of non-animal-derived nitrogen sources on HPV58 L1 production revealed a clear trend: the volumetric L1 yield increased primarily as a result of enhanced cell growth, whereas the specific yield remained stable or decreased. SYN6 supplemented with Hy-Express™ System II showed the highest volumetric and specific L1 yields, while SYN6 supplemented with HySoy, a more cost-effective nitrogen source, produced slightly higher L1 than the original SYN6 with minimal variation. Both nitrogen sources were, therefore, selected for further comparison in bioreactor-scale cultivation.

### Effect of cultivation temperature on cell growth and L1 protein production

Temperature plays an important role in yeast growth and recombinant protein production. *H. polymorpha* with its thermotolerant characteristics, can grow and produce recombinant protein within a range of 30–50 °C (Manfrão-Netto et al. 2019), unlike its counterpart *P. pastoris*, which generally requires lower cultivation temperatures (typically 20–25 °C) to achieve efficient recombinant protein production. Our results showed that increasing temperature generally improved cell growth, with the highest biomass (OD_660_) observed at 37 °C in media supplemented with HySoy. While statistical analysis showed no significant effect of temperature on volumetric L1 yield across all conditions, the highest production of HPV58 L1 protein was observed at 37 °C, which correlated with the condition that yielded the highest biomass.

This finding is notable when compared with the production of other HPV L1 proteins in *H. polymorpha*, where the optimal temperature for protein expression may differ from that for cell growth. For example, a recent study on HPV52 L1 production by Phimsen et al. (2024) reported that while the highest cell growth occurred at 37 °C, the maximum specific and volumetric protein yields were obtained at 30 °C. The trend observed for HPV52, where maximal biomass does not correspond to maximal protein production, clearly differs from our findings for HPV58. For HPV52, cultivation at 30 °C provides a more favorable balance between cell growth and protein production, while in case of HPV58 cultivation at 37 °C resulted in higher volumetric yield primarily due to increased biomass (Fig. 5). Such variation among closely related proteins (i.e., HPV52 and HPV58 L1 proteins) may arise from differences in folding efficiency or protein stability. Lower temperatures have been suggested to enhance HPV52 L1 stability and reduce degradation by host proteases (Phimsen et al. 2024). On the other hand, production of mammalian proteins in *H. polymorpha* has also been reported to benefit from cultivation at 37 °C, which supports proper folding and maintains biological activity (Manfrão-Netto et al. 2019). Therefore, the optimal temperature for recombinant protein production in *H. polymorpha* is highly protein specific. When comparing SYN6 with and without supplementation of HySoy, although a higher volumetric L1 yield was observed as a result of HySoy supplementation (Fig. 5b), a slight reduction in specific L1 yield (Fig. 5c) indicates that a portion of metabolic energy and resource may be directed toward growth rather than L1 production.

### Bioreactor-scale cultivation for enhanced L1 protein production

In bioreactor-scale cultivation, batch and fed-batch cultivation were applied with an aim to optimize HPV58 L1 production in *H. polymorpha*. Under batch mode, SYN6 supplemented with Hy-Express™ System II resulted in higher biomass (OD_660_ = 98 at 72 h), volumetric L1 yield (510 mg/L), and productivity (7.1 mg/L/h), which were markedly higher than those obtained with both SYN6 and SYN6 supplemented with HySoy (Fig. 6). These findings are consistent with reports demonstrating that complex nitrogen source supplementation can significantly enhance biomass and protein production efficiency in *H. polymorpha* (Mueller et al. 2013; Phimsen et al. 2024). While no noticeable enhancement was detected in flask-scale cultivation (Fig. 4), the improvement observed at the bioreactor-scale highlights the scale-dependent effects of medium optimization. Therefore, if possible, medium optimization should also be performed at the bioreactor scale, where pH and aeration can be precisely controlled.

In fed-batch (HCDC) mode, an exponential feeding with glycerol and Hy-Express™ System II showed the highest volumetric L1 yield, but a significant loss of the gene expression cassette was observed after 120 h of cultivation. Despite a shorter cultivation period, a similar degree of gene loss was also detected in batch cultivation with Hy-Express™ System II after 84 h. In contrast, exponential feeding with glycerol and HySoy maintained stable gene cassette retention throughout the entire cultivation period. This stability pattern observed with HySoy was consistent with the findings of Phimsen et al. (2024), who reported sustained expression of HPV52 L1 in *H. polymorpha* during HySoy-based fed-batch fermentation, with 100% of sampled colonies retaining the cassette after 120 h.

Gene loss was primarily reported for episomal plasmids when the selection pressure was removed (Bogdanova et al. 1995; Gatzke et al. 1995; Roggenkamp et al. 1986). In our case, although the recombinant L1 cassette was integrated at the *HARS1* site (an autonomously replicating sequence on the genome of *H. polymorpha*) with a low copy number (2–4 copies; unpublished data), gene loss was still observed in both batch and fed-batch cultivation using Hy-Express™ System II. The observed instability was hypothesized to result from the composition of Hy-Express™ System II, whose components are confirmed to include yeast extract, although the exact formulation is proprietary and not publicly disclosed. Yeast extract is known to contain uracil, which consequently reduces the selective pressure mediated by the *URA3* marker. While lower copy numbers generally favor increased genetic stability, reduced metabolic burden, and decreased risk of plasmid loss, our results indicate that insufficient selective pressure can promote instability of genome-integrated genes in *H. polymorpha*.

Among bioreactor-scale cultivations performed in this study (Fig. 7), fed-batch (HCDC) cultivation with HySoy under a constant feeding could maintain gene cassette stability and provided good volumetric L1 yield and productivity at a lower cost. Although supplementation of the SYN6 medium with Hy-Express™ System II during batch cultivation resulted in high volumetric yield, productivity and low cost, the observed gene cassette instability requires careful monitoring. In future work, this could be addressed by reducing the total amount of Hy-Express™ System II added, or by dividing the supplementation into multiple smaller pulses rather than a single large dose.

The trends of both volumetric and specific L1 yields in flask and bioreactor cultivations differed between HPV52, as reported by Phimsen et al. (2024), and HPV58, even though both L1 proteins were produced in the same *H. polymorpha* host. For HPV52, supplementation of complex nitrogen source promoted both cell growth and volumetric L1 yield. Based on the increase in specific yield, it appears that cells effectively utilized the supplemented nitrogen source for recombinant protein synthesis. Bioreactor-scale cultivation of *H. polymorpha* HPV52 demonstrated a remarkable improvement in productivity under HCDC, with the volumetric yield increasing 53-fold and productivity rising 27-fold compared with flask-scale cultivation (Phimsen et al. 2024). In contrast, the results from *H. polymorpha* HPV58 were entirely different, as specific L1 yield decreased under all conditions tested, suggesting that the supplemented nutrients were redirected toward cell growth rather than recombinant protein synthesis. The fact that increased biomass did not necessarily translate into enhanced protein production, as can be clearly seen from the volumetric L1 yield obtained from fed-batch cultivation with HySoy under constant and exponential feeding (Fig. 7), suggested that optimal rather than maximal growth may be more favorable for efficient HPV58 L1 production. Although HPV52 and HPV58 (with 89% similarity in amino acid sequences) were expressed in the same host, significant differences were observed in both optimal medium and bioreactor cultivation strategies, highlighting that even closely related proteins can impose distinct metabolic demands on the host, respond differently to cultivation conditions, and therefore require tailored medium composition and process strategies for optimal production. Although this study primarily focused on fermentation performance, cultivation temperature may also influence protein folding, stability, and the efficiency of virus-like particle (VLP) assembly. Therefore, further studies evaluating the effects of temperature on HPV58 L1 structural integrity and VLP formation will be important to ensure that improvements in production performance are accompanied by consistent product quality. In this regard, a scalable and robust downstream purification process for HPV58 L1 has recently been established, enabling the recovery of purified L1 protein suitable for structural and functional analyses (Kopitak et al. 2025). This platform will facilitate future investigations of the critical quality attributes of HPV58 L1 produced under different cultivation conditions.

## Conclusions

The optimized cultivation conditions for HPV58 L1 protein production in *H. polymorpha* were demonstrated in this study. Ferrous ion (Fe^2+^), in the form of ammonium iron (II) sulfate hexahydrate, and 1% of methanol were selected as the most optimal conditions and used in subsequent experiments. Cultivation temperature significantly influenced both cell growth and L1 protein production with 37 °C found to be most suitable for HPV58 L1 production. Supplementation of complex nitrogen sources helps stabilize the culture pH during flask-scale cultivation. Although the effect of complex nitrogen source supplementation was not noticeable in flask-scale cultivation, the results from batch and fed-batch bioreactor-scale cultivation evidently revealed the superiority of complex nitrogen source supplementation on HPV L1 production in *H. polymorpha* (1.7–5.5 folds increase in volumetric L1 yield and 1.7–3.2 folds increase in productivity).

## Supplementary Information

Below is the link to the electronic supplementary material.

Supplementary Material 1

## Data Availability

The datasets generated during and/or analyzed during the current study are available from the corresponding author on reasonable request.
