# Supplementary material for: Optimization of cultivation strategies for production of recombinant human papillomavirus type 58 major capsid protein L1 in Hansenula polymorpha
Source: Bioresour Bioprocess. 2026 May 25;13(1):75. doi: 10.1186/s40643-026-01059-8 (PMC13201806; doi:10.1186/s40643-026-01059-8)
Supplement: Supplementary file 1 — Supplementary Material 1 [file 40643_2026_1059_MOESM1_ESM.docx]

**Supplementary Materials**

**Optimization of cultivation strategies for production of recombinant Human Papillomavirus Type 58 major capsid protein L1 in *Hansenula polymorpha***

**Natsima Kopitak^1^, Wichittra Phimsen^1^, Kittipol Sripui^1^, Auntika Khunsom^1^, Natchanon Pongsuwichedsak^1^, Tatpong Boontawon^1^, Thantawat Theeranan^1^, Chuenchit Boonchird^1^, and Thunyarat Pongtharangkul^1,2^***

^1^*Department of Biotechnology, Faculty of Science, Mahidol University, Bangkok, Thailand*^2^*BioInnoTech, Faculty of Science, Mahidol University, Nakorn Pathom, Thailand*

**
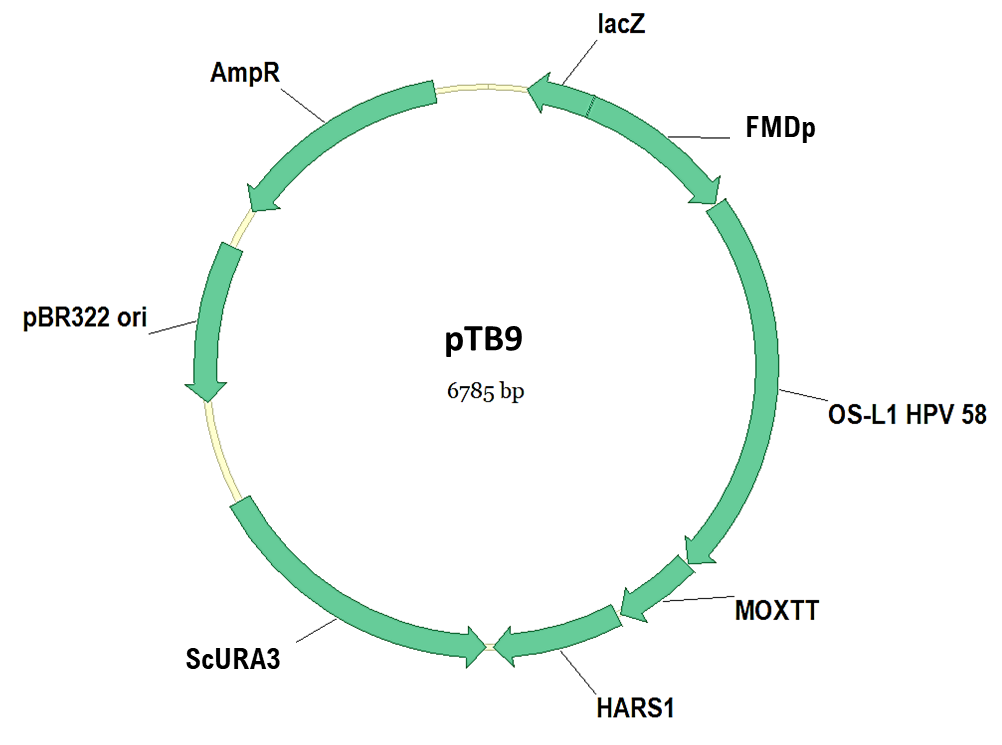
**

| **Figure S1.** | Plasmid map showing the L1 HPV58 gene and the complete expression cassette, including the *FMD* promoter, *MOX* terminator, integration at the *HARS*1 site, and the *URA*3 gene as a selectable marker. |
| --- | --- |

**Table S1.** Exponential feeding rate

| Feeding time,  t (h) | Feeding rate,  Ft (L/h) |
| --- | --- |
| 0 | 0.011 |
| 1 | 0.013 |
| 2 | 0.015 |
| 3 | 0.018 |
| 4 | 0.021 |
| 5 | 0.025 |
| 6 | 0.029 |
| 7 | 0.034 |
| 8 | 0.040 |
| 9 | 0.047 |
| 10 | 0.056 |
| 11 | 0.066 |
| 12 | 0.077 |
| 13 | 0.091 |
| 14 | 0.107 |
| 15 | 0.126 |
| 16 | 0.148 |

Note: Feeding rate was calculated from following parameters.

μ = 0.1625

Yx/s = 0.472 g-DCW/g-glycerol

X_0_ = 7.99 g-glycerol/L

V_0_ = 2 L

S_0_ = 400 g-glycerol/L

F_0_ = 0.0110 L/h

**Table S2.** Properties of non-animal derived, nitrogen sources evaluated in this study (BioTechnologies Technical Manual, Kerry, 2016)

| Properties | Hy-Express™ II | Hy-Express™ IV | HySoy | Hy-Yest 412 |
| --- | --- | --- | --- | --- |
| Source | Various non-animal sources | | Soy | Yeast |
| Digestion | Enzymatic | Enzymatic | Enzymatic | Enzymatic |
| Solubility (g/L) | 300 | >1,000 | >500 | <25 |
| Amino Nitrogen, AN (%) | 3.0 | 1.9 | 1.9 | 4.9 |
| Total Nitrogen, TN (%) | 13 | 9.8 | 9.1 | 10.9 |
| AN/TN ratio | 25 | 19.7 | 21.0 | 44.6 |
| Moisture (%) | 4.0 | 5.0 | 2.5 | 2.7 |
| Molecular weight distribution (%) | | | | |
| >10 kDa | 0.0 | 0.0 | 0.0 | 0.2 |
| 5-10 kDa | 0.1 | 0.1 | 0.3 | 0.1 |
| 2-5 kDa | 2.5 | 4.3 | 5.3 | 3.6 |
| 1-2 kDa | 10.4 | 15.2 | 16.4 | 14.4 |
| 500-1,000 Da | 18.2 | 21.1 | 23.5 | 17.2 |
| <500 Da | 68.8 | 59.3 | 54.6 | 64.4 |
| Minerals (%) | | | | |
| Ca | 0.04 | 0.10 | 0.20 | 0.2 |
| Fe | 0.0018 | 0.0053 | 0.006 | 0.0004 |
| Mg | 0.061 | 0.224 | 0.25 | 0.03 |
| P | 0.951 | 1.01 | 0.27 | 3.10 |
| K | 0.172 | 3.02 | 3.00 | 3.60 |
| Na | 1.66 | 2.17 | 2.70 | 1.70 |
| Amino acids (mg/g) | | | | |
| Alanine | 25 | 24 | 23 | 49 |
| Arginine | 24 | 27 | 37 | 31 |
| Aspartic acid | 37 | 59 | 74 | 63 |

**Table S2.** Properties of non-animal derived, nitrogen sources evaluated in this study (BioTechnologies Technical Manual, Kerry, 2016) (continued)

| Properties | Hy-Express™ II | Hy-Express™ IV | HySoy | Hy-Yest 412 |
| --- | --- | --- | --- | --- |
| Cysteine | 0 | 0 | 1 | 3 |
| Glutamic acid | 240 | 104 | 123 | 102 |
| Glycine | 20 | 16 | 22 | 27 |
| Histidine | 11 | 9 | 13 | 12 |
| Isoleucine | 23 | 15 | 20 | 29 |
| Leucine | 44 | 28 | 38 | 43 |
| Lysine | 22 | 32 | 39 | 46 |
| Methionine | 9 | 59 | 5 | 9 |
| Phenylalanine | 33 | 18 | 26 | 25 |
| Proline | 68 | 19 | 29 | 22 |
| Serine | 32 | 24 | 32 | 28 |
| Threonine | 20 | 18 | 22 | 28 |
| Tyrosine | 20 | 15 | 18 | 21 |
| Valine | 136 | 15 | 22 | 35 |
| Total amino acids | 763 | 482 | 542 | 573 |
| Total free amino acids | 72 | 145 | 63 | 255 |
| Free amino acids (%) | 9.4 | 30 | 11.6 | 44.5 |
